# Stem Cells, Self-Renewal, and Lineage Commitment in the Endocrine System

**DOI:** 10.3389/fendo.2019.00772

**Published:** 2019-11-08

**Authors:** Katia Mariniello, Gerard Ruiz-Babot, Emily C. McGaugh, James G. Nicholson, Angelica Gualtieri, Carles Gaston-Massuet, Maria Cristina Nostro, Leonardo Guasti

**Affiliations:** ^1^Centre for Endocrinology, William Harvey Research Institute, Bart's and the London School of Medicine and Dentistry, Queen Mary University of London, London, United Kingdom; ^2^Division of Endocrinology, Boston Children's Hospital, Boston, MA, United States; ^3^Harvard Stem Cell Institute, Cambridge, MA, United States; ^4^McEwen Stem Cell Institute, University Health Network, Toronto, ON, Canada; ^5^Department of Physiology, University of Toronto, Toronto, ON, Canada

**Keywords:** stem cells, development, self-renewal, regenerative medicine, plasticity

## Abstract

The endocrine system coordinates a wide array of body functions mainly through secretion of hormones and their actions on target tissues. Over the last decades, a collective effort between developmental biologists, geneticists, and stem cell biologists has generated a wealth of knowledge related to the contribution of stem/progenitor cells to both organogenesis and self-renewal of endocrine organs. This review provides an up-to-date and comprehensive overview of the role of tissue stem cells in the development and self-renewal of endocrine organs. Pathways governing crucial steps in both development and stemness maintenance, and that are known to be frequently altered in a wide array of endocrine disorders, including cancer, are also described. Crucially, this plethora of information is being channeled into the development of potential new cell-based treatment modalities for endocrine-related illnesses, some of which have made it through clinical trials.

## Introduction

Stem cells are endowed with the ability to self-renew and differentiate into various organ-specific cell types. They are mainly active during embryogenesis where complex autocrine, paracrine, and endocrine interactions govern their fate, proliferation, and gradual differentiation toward highly organized tri-dimensional organs. A growing number of evidence indicates that populations of stem cells are retained in most post-natal tissues (somatic or adult stem cells), where they exert essential functions throughout life, namely tissue maintenance/self-renewal, remodeling/plasticity in response to physiological demands as well as repair. By definition, adult stem cells have the ability to self-renew, however their differentiation potential is restricted to the array of specialized cell types corresponding to the organ in which they reside. The plasticity of the endocrine organs has been recognized only recently, and our understanding has been propellered by (i) the use of specific genetic mouse models, (ii) gene-discovery approaches for endocrine disorders, and (iii) reprogramming strategies to obtain functional endocrine cells. The acquired knowledge of the biology of endocrine organs is not only important for our understanding of pathological processes, but also for the potential application of cell-based therapies or restoration of stem cell function. In this review, the role of stem cells in the endocrine system will be covered, from the perspective of tissue development and their function in tissue maintenance and organ plasticity. Recent data showing potential to harness the properties of stem cells for clinical applications is also reviewed.

## The Pituitary Gland

### Endocrine Function in the Pituitary Gland

The pituitary gland is a small endocrine organ connected to the hypothalamus and together they form the hypothalamo-pituitary axis (HPA), which regulates vital physiological functions such as growth, reproduction, lactation, metabolism, and stress-responses (1). The pituitary gland can be separated into adenohypophysis (anterior pituitary), including both the anterior lobe (AL) and intermediate lobe (IL) derived from oral ectoderm, and the neurohypophysis (posterior pituitary) also known as the posterior lobe (PL) derived from neural ectoderm. In rodents the two lobes remain distinct and are separated by a cleft, with an epithelial lining known as the marginal zone (MZ). The PL is populated by the axonal termini of hypothalamic magnocellular neurons which release anti-diuretic hormone and oxytocin into the blood circulation. The AL develops from oral ectoderm and harbors five cell types: lactotrophs, producing prolactin (PRL); somatotrophs, which release growth hormone (GH); corticotrophs, which synthesize adrenocorticotrophic hormone (ACTH); thyrotrophs, secreting thyroid-stimulating hormone (TSH); and finally, gonadotrophs, which release luteinizing hormone (LH) and follicle-stimulating hormone (FSH). A further population of hormone secreting cells, melanotrophs, are found in the IL and are responsible for the synthesis of melanocyte-stimulating hormone (MSH).

### Key Pathways Guiding Pituitary Gland Development

**Figure 1:**
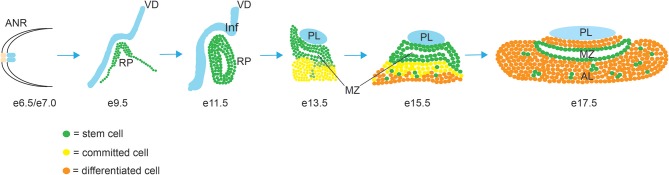
Morphogenesis of the mouse pituitary gland. Abbreviations: AL, anterior lobe; ANR, anterior neural ridge; IL, intermediate lobe; Inf, infundibulum; MZ, marginal zone; PL, posterior lobe; RP, Rathke's Pouch; VD, ventral diencephalon.

The development of the pituitary gland can be separated into three sequential steps, cell specification, cell lineage commitment and terminal differentiation (Figure 1). In mice, pituitary organogenesis begins at embryonic day (e) 8, with a thickening of a region of the oral ectoderm, known as the hypophyseal placode (HP) within the anterior neural ridge (ANR) and adjacent to the ventral diencephalon (VD). By e9, an epithelial invagination of the oral ectoderm, centered at the HP occurs forming a rudimental pouch known as Rathke's pouch (RP) (2). This process is directed by physical contact with the overlaying region of the VD known as the infundibulum, which eventually gives rise to the hypothalamic median eminence, the pituitary stalk and the PL (3). By e10.5 the infundibulum begins to evaginate toward the RP and tightly regulated apoptosis separates the RP from the underlying oral ectoderm (4). The lumen of the RP is surrounded by a highly proliferative epithelial layer of pituitary stem/progenitor cells (PSCs) (5). This cell population undergoes a rapid expansion between e11.5 and e13.5 during which the majority of endocrine cell precursors are generated (6). As these RP progenitors gradually exit the mitotic cycle, they express cell cycle inhibitors such as p57^KIP2^ and p27^KIP1^ and lose their epithelial characteristics in order to give rise to distinct pituitary cell types (7). By e14.5 PSCs are committed to one of the three endocrine lineages (expressing transcription factors T-box Factor 19, Pituitary (Tpit) (8), or POU domain, class 1, transcription factor 1 (Pit1)(9) or Steroidogenic Factor 1 (Sf1) (10), and as they begin to differentiate they migrate ventrally and laterally away from the RP lumen, forming the bulk of the AL, with the dorsal progenitors of the RP forming the IL. The residual luminal space of the RP, known as the cleft, and the periluminal MZ constitutes a stem cell niche where multipotent PSCs are maintained into adulthood (11–13). Terminal differentiation culminates shortly after birth when, in rodents, the pituitary gland undergoes a sustained perinatal period of proliferation and growth (14).

Patterning of the developing embryo and induction of the pituitary primordium are regulated by a complex array of sequentially expressed signaling molecules and morphogens. Together, these act to demarcate different regions of the VD (15) and control the developmental induction of the pituitary gland. Bone morphogenetic protein-4 (Bmp4) is expressed and secreted from the VD from e8.5; there it functions as an essential extrinsic requirement for RP formation, and its expression is maintained in the infundibulum up to e14.5 (16–19). Through induction of T-box transcription factor 2 (Tbx2), Bmp4 represses the expression of the morphogen sonic hedgehog (Shh) (20) and opposing gradients of Bmp4 and Shh define the infundibular region of the VD ensuring correct positioning of the RP. From e10.5 Bmp2 is expressed in the developing RP and is essential for RP maintenance and progenitor proliferation, before expression is lost by e14.5 (6, 16, 17). Fibroblast growth factor (Fgf)- 8, -10, and -18 are also expressed in the developing VD, appearing shortly after Bmp4 and maintained until e14.5 (17, 19, 21). Fgfs secreted by the VD activate the MAPK signaling pathway to promote the maintenance and proliferation of the dorsal region of the RP (6). Shh expression in the VD depends on SRY-box transcription factor 2 (Sox2) and Sox3 (22), and ensures the correct patterning of the region. The reciprocal inhibition of Shh and Bmp4 is required for correct infundibular positioning and induction of the RP. Consequently, ablation of Shh expression in the VD has been shown to cause altered expression of Bmp4, Wnt5a, and Fgf8 and a complete arrest in pituitary formation from the early stages of development (23, 24). Within the RP itself, Shh signaling is involved in progenitor proliferation as revealed by conditional deletion of its downstream transcriptional repressors Gli2 and Gli3 (25). Notch signaling in pituitary development appears important for infundibular morphogenesis, as mice null for the known target of Notch, Hairy and Enhancer of Split-1 (*Hes1*) have reduced evagination and disrupted development of the posterior lobe (26–29). Within the RP, Notch signaling is initially widespread and later restricted to the MZ (29, 30). There it promotes progenitor proliferation and maintenance (31), suppresses melanotroph and corticotroph differentiation (32) and promotes the emergence of the Pit1 lineage through integration with the transcription factor Homeobox Protein prophet of Pit1 (Prop1) (29, 33).

Wnt5a is expressed in both the VD and RP from e9.5 to e12.5 and is necessary for correct VD patterning, and indirectly for RP induction via non-canonical pathway (34). Wnt4 also signals via the non-canonical Wnt pathway, is expressed exclusively in the RP, and appears to function in cell commitment since its deletion reduces the expression of Pit1 resulting in fewer somatotrophs, lactotrophs, and thyrotrophs (34). Canonical Wnt/β-catenin also plays an important role in pituitary development, and conditional gain or loss of function studies of β-catenin within the VD showed its role in regulating the expression of Fgf8, necessary for normal RP development (35). Within the RP, β-catenin has a role in Pitx2 activation stimulating progenitor proliferation (36) and later binds Prop1 and is necessary for the emergence of Pit1 lineage of endocrine cells (37). Further downstream of Wnt signaling, the transcription factor binding partners of β-catenin, Transcription factor Tcf3, Tcf4, and Lymphoid Enhancer Binding Factor 1 (Lef1), also play a role in pituitary development. Tcf3 acts as a repressor of the Wnt/β-catenin pathway in the anterior forebrain (38) and is essential for the development of the HPA in both human and mice (39). Tcf4 genetic ablation leads to an increase in early progenitor proliferation with increased and prolonged expression of Prop1, which can lead to aberrant tissue growth and tumor formation if not down-regulated (40–42). Genetic ablation of Lef1 does not have similarly pronounced effects, but its inhibition reduces Pit1 expression indicating a potential role as a repressor of pituitary differentiation (37, 43). The Wnt/β-catenin pathway is important in PSC proliferation and maintenance and deregulation of this pathway lead to stem cell-derived pituitary tumors. Activating mutations in β-catenin drive adamantinomatous craniopharyngioma both in mouse and humans (44, 45) and PSC Sox2^+^ cells have been shown to be the tumor initiating cells that are responsive to oncogenic β-catenin.

**Figure 2:**
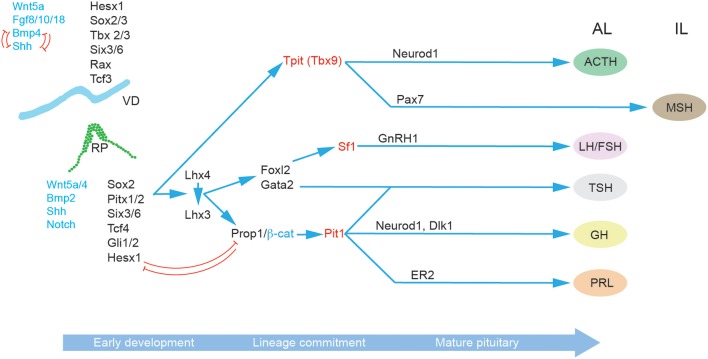
Molecular regulation of pituitary gland development. A succession of transcription factors (black) and signaling molecules (blue) determine the establishment of RP and the subsequent lineage specification and differentiation in the progenitor cells of the developing pituitary hormone-secreting cell types characteristic of the mature anterior pituitary gland: corticotrophs (ACTH), gonadotrophs (FSH and LH), thyrotrophs (TSH), somatotrophs (GH), and lactotrophs (PRL). The key lineage commitment makers are highlighted in red. Arrows indicate upstream relationships in molecular signaling pathways, not necessarily direct activation. Red T-bar arrows denote repressive relationships. Abbreviations: ACTH, adrenocorticotropic hormone; AL, anterior lobe; FSH, follicle-stimulating hormone; GH, growth hormone; IL, intermediate lobe; LH, luteinizing hormone; MZ, marginal zone; PL, posterior lobe; PRL, prolactin; RP, Rathke's pouch, VD; Ventral diencephalon.

In addition to the role of morphogens and signaling pathways, the spatiotemporal expression patterns of transcription factors during pituitary development have also been extensively studied, particularly in the context of congenital forms of hypopituitarism (Figure 2) (46). The paired-like homeodomain transcription factor Homeobox Expressed in ES cells 1 (Hesx1) functions as a transcriptional repressor through its interaction with the transcriptional corepressor Transducin-Like Enhancer of Split 1 (Tle1) and is an important regulator of forebrain development (47). It is also crucial for early pituitary development, with Hesx1^−/−^ mice showing multiple clefts and over proliferation (48). Importantly, in the RP Hesx1 represses Prop1 expression until e13.5 (48) when it is reciprocally downregulated by the Prop1/β-catenin complex (37). Hesx1 also acts as a repressor of the Wnt pathway, and it has been suggested that de-repression of the Wnt pathway in the anterior neural plate and RP underlies the phenotype of Hesx1^−/−^ mice (48, 49). The closely related Sine Oculis homeobox (Six) transcription factors Six3 and Six6 are expressed in both the VD and RP, with Six6 expression maintained in the adult pituitary. Knockout studies have revealed that both transcription factors are involved in the regulation of progenitor proliferation, with Six6 acting a repressor of the cell cycle inhibitor p27KIP1 (50), and Six3 serving as a repressor of Wnt/β-catenin signaling (49). The paired homeodomain proteins, Pitx1 and Pitx2, are two additional important regulators of pituitary development expressed in the RP where they function redundantly in the maintenance of RP progenitors (4) and later play a role in thyrotroph function (51). Three different members of the LIM-homeodomain transcription factors (Lhx2, 3 and 4) are expressed during pituitary development. Lhx2 is expressed throughout the RP and VD, and appears to function in formation of the infundibulum, but is not involved in cell differentiation (52). By contrast Lhx3 and Lhx4 are expressed from e9.5 in the RP and are redundantly required for progenitor maintenance, and later at e14.5 Lhx4 is downregulated whilst Lhx3 expression is required for endocrine differentiation and maintained into adulthood (53). As stated above, Sox2 and Sox3 are expressed in the VD where they activate the expression of Shh (22) and of Six3/Six6 proteins (54, 55). Sox3 loss of function mutations can result in mild hypopituitarism (56), as can Sox2 haploinsufficiency (57). In both *Sox3*^−/^^−^ and *Sox2*^+/^^−^ mice the RP is bifurcated, and at least for *Sox3*^−/^^−^ mutants this has been associated with expanded Bmp4 and Fgf8 domains in the VD (57, 58). This is likely a consequence of downregulation of Shh (22) and perhaps Six3/6 also (54, 55). Prop1 represents the earliest pituitary specific marker; it is first expressed at e10, and maintained throughout development in the Sox2^+^ progenitor cells, before rapid post-natal downregulation in all but a few Sox2^+^ PSCs (59–61). Prop1^−/−^ mice have reduced Pit1 expression, and prolonged *Hesx1* expression resulting in the loss of somatotrophs, lactotrophs, and thyrotrophs (59, 60, 62). An important role of Prop1 is the regulation of the epithelial-to-mesenchymal transition as progenitor cells migrate away from the residual RP lumen and begin to undergo differentiation. In the absence of Prop1, progenitors fail to populate the anterior lobe resulting in a dysmorphic pituitary gland by e14.5 (63, 64).

Progenitor endocrine cell lineage commitment is defined by the expression of three essential transcription factors Pit1, Tpit, and Sf1 (Figure 2). The process of differentiation relies on the activity of at least two epigenetic regulators, the histone demethylase Lsd1 (65), and the zinc finger protein Insm1 (66). Pit1 expression is activated by Prop1, in complex with β-catenin (37) and is required for the differentiation as well as the expansion and survival of lactotrophs, somatotrophs and thyrotrophs (67, 68). Somatotrophs are further specified by Neurod4 (29), and the Notch ligand Delta-Like homolog 1 (Dlk1) (69). In contrast lactotrophs are predominately specified by estrogen signaling (70). Thyrotrophs can first be identified by the expression of the transcription factor Forkhead Box L2 (Foxl2) and then α-Glycoprotein Subunit (αGSU) (19). Both are also expressed in gonadotrophs. Subsequently, Gata2 is expressed which can activate the expression of Chromogranin-A (Cga) (71). Gonadotrophs are broadly similar to thyrotrophs in terms of their expression of lineage commitment markers but can be differentiated by their expression of Gonadotropin Releasing Hormone Receptor Gnrhr (72) and later Sf1 which promotes the expression of Cga, Fsh and Lh (73). Corticotrophs and melanotrophs emerge from the Tpit (Tbx19) lineage (74, 75) which are further defined by their expression of the transcription factors Neuronal Differentiation 1 (NeuroD1) (76) and Paired Box 7 (Pax7) (77), respectively.

### Stem Cells in the Developing and Adult Pituitary Gland

The past decade has seen a great deal of interest in the characterization of PSCs and their function through development to the maintenance of the adult gland, under normal physiological conditions, periods of endocrine stress or in pituitary disease (78–83). They are primarily identified by their expression of Sox2, which drives rapid proliferation in the lumen of RP during early development (84). By e13.5 the surge in pituitary precursor proliferation subsides and Sox9 is expressed alongside Sox2 in a subpopulation of PSCs (85). The AL also harbors a secondary stem cell niche with clusters of Sox2^+^ PSCs scattered through the parenchyma (46, 86). Functional analysis of PSCs from these two different niches did not reveal obvious differences (87). Intriguingly these two disparate populations of PSCs appear to be physically interconnected to form a three-dimensional network, an architectural feature that hints at some, as of yet undescribed, concerted function (88).

Building upon early *in vitro* studies (12), more recent *in vivo* lineage tracing studies have demonstrated the multipotency of Sox2/Sox9^+^ embryonic and adult PSCs and their contribution to tissue homeostasis (85, 89). Intriguingly, under normal physiological conditions adult PSCs are highly quiescent and largely inactive (15, 17, 19, 23), which may reflect the low tissue turnover rate of adult pituitary cells, relative to tissues with more active stem cell pools (24); this questions the notion of tissue maintenance as their function (17), particularly since major depletion of adult PSCs did not affect tissue homeostasis (90). More likely their primary function is to provide the organ with plasticity and some regenerative capacity. Genetic ablation of different endocrine cell populations induces PSCs activation and replenishment of ~60% of the targeted hormone secreted cell type (25–28). However, this regenerative capacity is limited, as it tails off rapidly with age (28) and there is at least some contribution from endocrine cell proliferation and trans-differentiation (26). Perhaps more importantly, PSCs are also able to respond to physiological demand under periods of endocrine stress: for instance, experimental adrenalectomy leads to increases in Sox2^+^ PSCs-derived corticotrophs and gonadotrophs (19, 29). Interestingly, if instead corticotrophs were depleted gradually, then the progenitor pool was not activated (30), suggesting that the loss of signal from the end organ may be required for PSCs mobilization (29). During pregnancy, the number and activity of lactotrophs rise in an expansion partly driven by estrogen (31) and indeed treatment of male mice with oestradiol causes a sharp rise in Sox2^+^ PSC proliferation, a finding also observed in experimental gonadodectomy (19). The heightened activity of PSCs during the neonatal expansion of the gland and production of new endocrine cells is also clear, and they show increased proliferation as well as multipotent differentiation capacity (91). The potential involvement of PSCs in the subtler changes to the gland that occur during puberty and sexual maturation is logical, but as of yet unproven.

### Stem Cells and Regenerative Medicine in the Pituitary Gland

Recent advances in the *in vitro* recapitulation of pituitary development highlight the potential of cell-based therapies to revolutionize the treatment of hypopituitarism, which is defined by the failure to secrete one or more pituitary hormones, and typically requires lifelong hormone replacement therapy. Pioneering work by Suga et al. reported the induction of self-organizing RP-like structures from mouse ES-cells, which contained corticotrophs and somatotrophs and were capable of rescuing systemic glucocorticoid level in hypopituitary mice (92). Mimicking pituitary development, their protocol involves the induction of adjacent layers of non-neural head ectoderm and hypothalamic neuroectoderm, which a follow up study showed was also applicable to human embryonic stem cells (ESCs) (93). Using an alternative approach, Dincer et al. were able to induce a placodal fate in adherent hESCs cultures, ultimately producing functional corticotrophs, that secreted ACTH after subcutaneous implantation in mice (94). Preliminary attempts at generating pituitary organoids from adult mouse PSCs have been performed, though to date, these have lacked the degree of self-organization, and functional hormone release achieved by their ESCs-derived counterparts (95). Interestingly, in all strategies ACTH-secreting cells are the predominant differentiated endocrine cell type produced. Future work will likely focus on the targeted generation of other hormone secreting cells, and move toward orthotopic transplants to investigate the degree to which transplanted cells can integrate into the regulatory circuitry governing physiological hormone secretion.

## Adrenal Cortex

### Endocrine Function and Key Pathways Guiding Adrenal Cortex Development

The adrenal cortex is essential for life. It is the primary site of steroid synthesis, producing glucocorticoids under the control of the HPA and mineralocorticoids under the control of the renin-angiotensin-aldosterone system (RAAS). Glucocorticoids regulate glucose metabolism, inflammation, immune responses, muscle and skeletal mass as well as cognition, well-being and memory, while mineralocorticoids control extracellular fluid volume and sodium homeostasis, and hence have an important influence on blood pressure.

**Figure 3:**
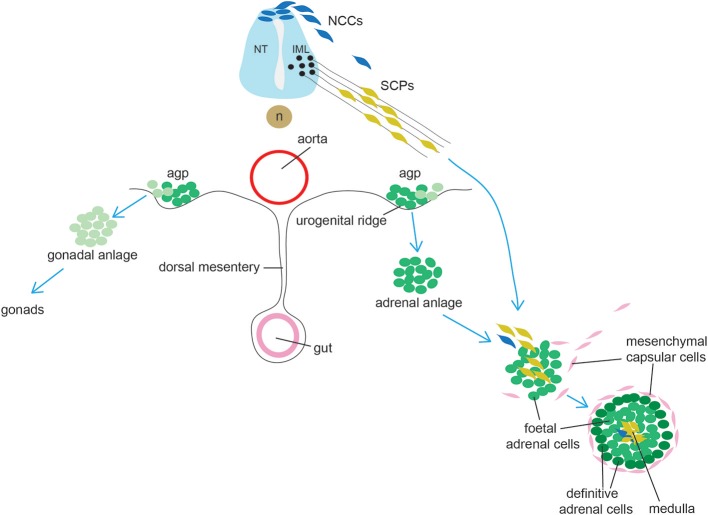
Schematic representation of adrenal gland development. Cells from the adrenogonadal primordium (agp) form the adrenal and gonadal anlage. The adrenal anlage is invaded by migrating medullary progenitors who derive from early migrating neural crest-derived cells (NCCs, a minority in mice) and from late migrating Schwann cell precursors (SCPs). Concomitantly, the adrenal is encapsulated by mesenchymal cells. During late embryogenesis, definitive adrenal cells appears and will substitute fetal adrenal cells. IML: intermediolateral column (IML); NT, neural tube; n, notochord.

The adrenal cortex originates from a group of cells within the dorsal coelomic epithelium at ~e9.0 in mice and 3–4 weeks in humans (Figure 3). These cells form the so-called adrenogonadal primordium (agp) and express the master regulator of adrenocortical differentiation and function, namely Steroidogenic factor-1 (Sf1, encoded by Nuclear Receptor Subfamily 5 Group A Member 1 -*Nr5a1*) (96). Sf1^+^ cells delaminate from the coelomic epithelium and invade the overlying mesonephric mesenchyme. The agp then separates forming the adrenal anlagen migrating dorsomedially and the gonadal anlagen, which settles dorsolaterally. Genetic and molecular evidence have demonstrated that the transcription co-factor Cbp/P300-Interacting Transactivator 2 (Cited2) interacts with the transcription factor Wilms Tumor 1 (Wt1) to stimulate expression of Sf1 in the agp prior to the separation between gonadal and adrenal primordia (97). The adrenal primordium is then invaded by migratory neural crest- and Schwann cell precursors-derived cells that will form the neuroendocrine medulla (see section on adrenal medulla). Subsequently the gland becomes encapsulated by mesenchymal cells. The cortex is composed of fetal adrenal cells that are established before the outer definitive adrenal population emerges between the capsule and fetal adrenal. Functional zonation is completed around birth. A crucial lineage relationship between fetal adrenal cells and adrenal capsular cells to the differentiated adrenal cortex was determined using specific Cre lines permitting the identification of cells that have at some time actively expressed *Nr5a1* under control of the fetal adrenocortical-specific enhancer (*FAdE*), an essential element in driving and maintaining Sf1 expression in the fetal cortex. These experiments indicated that a subset of capsular cells are indeed descendants of fetal adrenocortical cells that once expressed Nr5a1 (98).

### Stem Cells and Self-Renewal in the Adrenal Cortex

**Figure 4:**
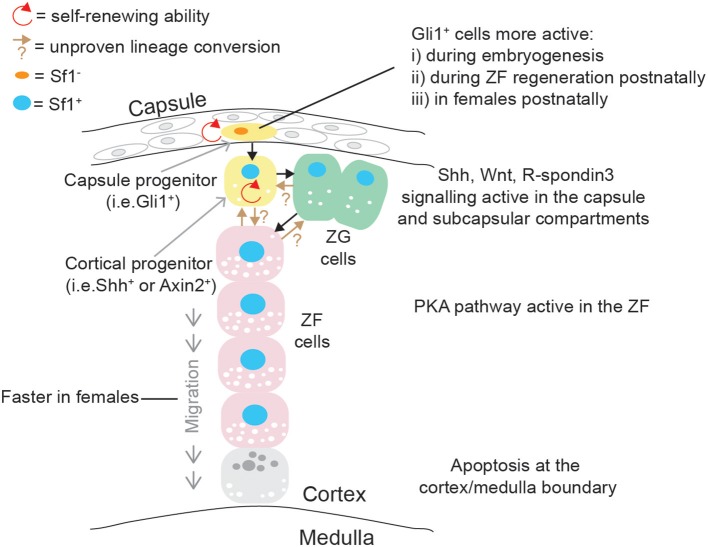
Schematic representation of post-natal adrenal cortex centripetal streaming and self-renewal in mice. Gli1^+^ cells in the capsule can give rise to Sf1^+^/Shh^+^ cortical cells: both are self-renewing adrenocortical progenitor cell populations. Shh^+^ cells can become ZG cells, and ZG cells can lineage convert to ZF cells, which migrate centripetally. Direct differentiation between Shh^+^ cells and ZF is probably occurring in parallel. These differentiation events are governed by pathways mostly active in the capsular/subcapsular region, while apoptotic figures are observed at the cortex/medulla boundary in senescence cells.

The adrenal cortex undergoes a self-renewal process and important paracrine effectors supporting a dynamic centripetal streaming of adrenocortical cells have been identified with the use of specific mouse transgenic models (99). Adrenocortical self-renewal in the experimental animal relies on the differentiation of at least two cell populations of progenitor cells, located in capsular and subcapsular compartments (Figure 4). It was shown that Shh is expressed in Sf1^+^ but relatively undifferentiated cortical cells in the subcapsular region of the mouse (100, 101) and rat (102) adrenal starting from e12.5 and e13.5, respectively. Capsule cells transduce the Shh signal, and lineage-tracing studies have shown that Gli1^+^ capsular cells delaminate into the cortex, lose their responsiveness to Shh, and become Shh^+^/Sf1^+^ progenitor cells; they then proceed to become fully mature steroidogenic cells forming the distinct histological and functional layers: zona glomerulosa (ZG, secreting aldosterone and expressing aldosterone synthase, encoded by *Cyp11b2*) and zona fasciculata (ZF, secreting glucocorticoids, expressing 11βhydroxylase, encoded by *Cyp11b1*) (100). Capsular Gli1^+^ cells and subcapsular Shh^+^ cells are therefore two interconnected types of adrenocortical progenitor cells; recently however it has been shown that the relative impact of capsular and subcapsular progenitor cells in generating new steroidogenic cells is extremely unbalanced post-natally with cortical Shh progenitor cells being preponderant in generating steroidogenic cells compared to the capsular Gli1 population (103).

Another player in the gland self-renewal is paternally expressed Dlk1/Preadipocyte Factor-1 (Pref1), a cleavable single-pass transmembrane protein and a member of the Notch/Delta/Serrate family. A number of experimental evidence suggest that Dlk1 may be involved in adrenocortical differentiation: (i) Dlk1 is expressed in Shh^+^/Sf1^+^ progenitor cells in rat adrenals (104), (ii) Dlk1 regulates *Gli1* levels in H295R cells, possibly through the secreted ectodomain Dlk1 and in a β1-integrin dependent fashion (104), (iii) its expression was found to be inversely correlated to the differentiation status of the ZG following remodeling of RAAS rats (104), (iv) its potential cross-talk with subcapsular Fgf signaling, as *Fgfr2IIIb* knock-out mice showed hypertrophic capsule and absence of capsular Dlk1 expression (105), and (v) its rapid disappearance after adrenal enucleation in rats and reappearance once zonation is restored (106). These data suggest that Dlk1 might be a negative regulator of adrenocortical differentiation, similarly to its well-established role in inhibiting adipogenesis (107). Interestingly, while Dlk1 is expressed in the subcapsular region of rat (104) and human (108) adrenals, it is mostly expressed in capsular cells in mice (105, 109). It is not currently known whether Dlk1 is co-expressed with Gli1^+^ progenitor cells in the capsule, or whether Dlk1 and Gli1 mark two different populations.

The lineage relationship between fully differentiated ZG and ZF cells during post-natal life and during regeneration was established with the development of a specific mouse model where Cre recombinase was targeted to the *Cyp11b2* genomic locus (110). Genetic lineage tracing with these mice demonstrated that ZG cells can lineage convert to ZF cells in a Sf1 dependent manner, and these cells can mark the whole ZF in a period of 12 weeks, suggesting a relatively slow self-renewing process in the mouse adrenal cortex. However, as Cyp11b2 knock-out mice are still able to generate ZF cells, alternative and/or facultative cell sources active in sustaining ZF self-renewal (and therefore crucial in preserving homeostatic cortisol levels) must be present, one example of such could be a subset of capsular/subcapsular progenitors able to directly differentiate toward a ZF identity.

Compelling evidence of the importance of other pathways in adrenocortical growth, self-renewal and zonation, has also been provided; for example, targeted disruption of β-catenin in Sf1^+^ cells resulted in an impairment of adrenal cortex development and maintenance in mice; this phenotype was even more pronounced when a Cre transgene was expressed at high levels, resulting in adrenal aplasia (111). Conversely, constitutive β-catenin activation induced ZG adrenal hyperplasia which ultimately led to adrenal cancer development in mice (112). Wnt-responsive cells were found to be Shh^+^ progenitor cells as well as differentiated, steroidogenic cells of the ZG, but not the ZF, and rarely cells that were actively proliferating. *In vitro* experiments also demonstrated that stimulation of β-catenin signaling caused decreased corticosterone release; this was corroborated by not only a reduced expression of steroidogenic genes such as *Cyp11a1, Cyp11b1, Star*, and *Mc2r*, but also by a diminished Sf1 expression and Sf1 occupancy on steroidogenic promoters. Interestingly, Coiled-Coil Domain Containing 80 (Ccdc80) was found to be a novel β-catenin-regulated gene in adrenocortical cells, and secreted Ccdc80 could partially phenocopy suppression of steroidogenesis induced by β-catenin, in a Sf1-independent fashion (113). Wnt4 is key activator of the pathway in the cortex and knock-out experiments in mice demonstrated that capsular R-spondin3, a secreted protein and a known positive regulator of Wnt/β-catenin pathway, induces Wnt4 and Shh expression within steroidogenic cells in the subcapsular compartment (114) and that its action is strongly antagonized by protein kinase A (PKA) activation, resulting in inhibition of ZG differentiation. PKA stimulation was able to increase inactivating and decrease activating β-catenin phosphorylation in adrenocortical cells *in vivo*. Therefore, it was suggested that PKA activation in the ZF is a key driver of Wnt inhibition and lineage conversion of cells to a ZG identity. The same authors provided evidence that constitutive PKA activation was able to inhibit β-catenin-induced ZG adrenal hyperplasia and subsequent tumorigenesis *in vivo* (115). Constitutive PKA activation, which was achieved by genetic deletion of the critical component Protein Kinase cAMP-Dependent Type I Regulatory Subunit Alpha (Prkar1a) was also found to be crucial for conversion of ZF cell to a zona reticularis (ZR)-like phenotype, seemingly via lineage conversion of the innermost ZF cells; interestingly this process was found to be sexually dimorphic as testicular androgens were shown to increase adrenocortical Wnt signaling (antagonizing PKA), leading to slower adrenocortical cell turnover and delayed ZR appearance whereas gonadectomy sensitized males to hypercorticism and ZG-like formation (116). More recently, a thorough study of adrenocortical self-renewal in mice shed more light on this sexually dimorphic phenomenon: genetic lineage tracing was achieved using Axin2 mouse model; Axin2 has been shown to reliably act as a readout for Wnt-responsive cells, and, as such, it is a frequently-used marker of functional stem cells. By comparing male and female mice, they found that female mice had significantly higher proliferation as well as turnover than males; moreover, in females but not males, the capsule Gli1^+^ population was found to be more active in generating new steroidogenic cells post-natally. Interestingly, proliferation rates, cortex turn-over and recruitment of capsular Gli1^+^ cells was enhanced in males following orchiectomy, suggesting that androgens might inhibit full recruitment of some adrenal cortex stem cell compartments; this was further corroborated by showing inhibition of Gli1^+^ cells activation in ovariectomised females treated with dihydrotestosterone. This data is important as it might explain the biology behind the higher incidence of adrenal diseases in females (117).

Important factors involved in adrenal cortex differentiation and self-renewal under physiological conditions have also been discovered by assessing mutation and changes in gene expression in adrenocortical tumors. Two examples are the histone methyltransferase Enhancer of Zeste Homolog 2 (Ezh2), the most deregulated epigenetic factor in adrenocortical carcinomas (118) and the transmembrane E3 ubiquitin ligase zinc and ring finger 3 (Znrf3), a known Wnt inhibitor which is frequently inactivated in adrenocortical carcinomas (119). Ezh2 was found to be an important epigenetic factor ensuring the unidirectionality of differentiation events from ZG to ZF. Targeted inactivation of Ezh2 in mouse adrenal cells was achieved through the use of a Sf1Cre line crossed to a floxed Ezh2 allele; these mice had hypoplastic adrenal glands and developed primary glucocorticoid insufficiency (low corticosterone, high ACTH, normal aldosterone in female mice) with blunted ZF differentiation (decreased *Cyp11b1* mRNA expression), suggesting that Ezh2 is a key regulator of ZF differentiation and identity. This suggestion was proved by further experimental data showing that Ezh2 not only programmed adrenocortical cells to respond to ACTH via PKA signaling, but also inhibited accumulation of capsular/pericapsular Gli1^+^ and Wt1/Gata4^+^ spindle-like pericapsular cells. Interestingly, these Gli1^+^ and Wt1/Gata4^+^ fibroblastic-like cells, commonly seen in aged or gonadectomised mice of specific backgrounds, were found to be derived from steroidogenic (Sf1^+^) cells through a mechanism involving dedifferentiation, rather than direct amplification of capsular cell populations (120).

Znrf3 was found to be expressed in both ZG and ZF cells in mice; adrenocortical-specific loss of Znrf3, achieved through the use of both Sf1Cre and Cyp11b2Cre lines crossed to a floxed Znrf3 allele, developed adrenal hyperplasia in the ZF in a ACTH-independent manner with loss of normal adrenocortical architecture; this phenomenon was found to be dependent on Wnt signaling as genetic inactivation of Znrf3 together with Porcupine (a key enzyme required for Wnt ligands maturation and activity) displayed normal adrenal cortex architecture and reduced ZF hyperplasia. The authors also found that Wnt4, normally highly expressed in the ZG with a decreasing gradient into the outer ZF, lost this characteristic expression pattern and instead displayed moderate-level expression throughout the entire ZF. Interestingly, this pattern was also altered for β-catenin protein expression, as well as *Axin2* mRNA, strongly suggesting that loss of Znrf3 leads to increased Wnt/β-catenin in the ZF promoting hyperplasia (121).

### Stem Cells and Regenerative Medicine in the Adrenal Cortex

There is an undeniable case for stem cell regeneration therapy in adrenal insufficiency, however it is still in its infancy. Initial experiments showed the ability to obtain a steroidogenic lineage when Sf1 was forced-expressed in hESCs (122). Since then, others have reported the conversion of mouse and human ESCs, mesenchymal stem cells and inducible pluripotent stem cells (IPSCs) into adrenocortical-like cells, all by over-expressing Sf1 [reviewed in (123)].

Our laboratory has devised a technology for the generation of steroidogenic-like cells via reprogramming of skin-, blood- and urine-derived cells in humans. Reprogramming was achieved via forced expression of Sf1 through lentiviral delivery, together with the activation of the protein kinase A (PKA) pathway and in the presence of luteinising hormone releasing hormone (LHRH). These reprogrammed cells had ultrastructural features resembling steroid-secreting cells, expressed steroidogenic enzymes and secreted steroid hormones in response to physiological and pharmacological stimuli. They were viable when transplanted into the mouse kidney capsule and intra-adrenal. Importantly, the hypocortisolism observed in cells derived from patients with adrenal insufficiency due to congenital adrenal hyperplasia was rescued by expressing the wild-type version of the defective disease-causing enzymes. This study provided for the first time an effective tool with many potential applications to study adrenal biology and pathobiology in a personalized manner and opened up avenues for the development of precision therapies (124). The main obstacle to a clinical application of the strategies described above is the absence of a protocol which allows derivation of (i) proper adrenocortical-like cells from pluripotent stem cells or somatic cells without overexpression of Sf1, and (ii) a cell population able to self-renew similarly to the cortex.

## Adrenal Medulla

### Endocrine Function of the Adrenal Medulla

The adrenal medulla is the inner part of the adrenal gland and is mainly responsible for the synthesis and secretion of catecholamines, such as epinephrine (adrenaline) and norepinephrine (noradrenaline), both derived from the aminoacidic tyrosine and stored in vesicles prior to secretion. The main cell type of the adrenal medulla is the chromaffin cell (or pheochromocytes), named as such because of the affinity of catecholamines for chromium salts. The adrenal medulla is highly innervated by preganglionic sympathetic fibers. Epinephrine and norepinephrine are responsible for the execution of the fight-or-flight response of the sympathetic nervous system; such response involves (i) an increase in blood pressure via binding and activation of α1 receptors on vascular smooth muscle cells (resulting in vasoconstriction and increased blood flow to muscles and brain); (ii) an increase in the heart rate and contractility, (iii) a relaxation of smooth muscles in the airways (via β2-adrenoreceptors, to increase breathing), (iv) an increase in glycaemia via activation of the glycogenolysis pathway concomitant to stimulation of glucagon secretion via β2 receptors and decreasing of insulin secretion via α2 receptors in the Islets of Langerhans.

### Key Pathways Guiding Adrenal Medulla Development

Until very recently, chromaffin cells were thought to be direct derivates of neural crest, with a stream of neural crest-derived cells migrating and committing to a common sympathoadrenal lineage ending up in the vicinity of the dorsal aorta, where they would proliferate and continue migrating either ventrally (cells forming the adrenal medulla) or dorsally (cells forming the sympathetic ganglion) (125, 126). In 2017 Furlan et al., using genetic lineage tracing approaches and genetic ablation, convincingly showed that the majority of chromaffin cells derive from a specific cell type, termed Schwann Cell Precursor (SCP) (127) (Figure 3). SCPs are the earliest well-defined glial-like cell population during peripheral nervous system development (the radial glial being their corresponding identifiable precursor in the central nervous system) and known multipotent stem cells which can differentiate and generate different cell types, such as the parasympathetic nervous system (128, 129). SCPs themselves are a neural crest derivates which have undergone an extensive change in gene expression with many glial-associates genes (which are also expressed in Schwann cells) being activated. SCPs appear in mouse at ~e12.5; later in development, they are also able to generate the so-called immature Schwann cells, which differentiate to form Myelin- and Remak- (non-Myelin) Schwann cells. SCPs have an intimate association with neurons and their processes and are extremely dependent on axonal signals for both migration, survival and differentiation, a feature that is not shared by neural crest cells, which migrate more freely. Over time, it has become clear that SCPs could generate direct derivates which were of a different lineage to Schwann cells, such as endoneurial fibroblasts (130), parasympathetic ganglia (128, 129), melanocytes (131), and mesenchymal cells giving rise to odontoblasts and tooth pulp cells (132). Furlan et al. traced SCPs using neural crest and glial-specific inducible Cre lines [Sox10 and the myelin component Proteolipid Protein 1 (Plp1)]. Injection of tamoxifen at e11.5 followed by analysis at e17.5 showed that at least half of chromaffin cells in the adrenal medulla derived from SCPs. The contribution of nerve-associated SCPs was further corroborated by genetic ablation of SCPs with diphtheria toxin subunit A, resulting in a significant depletion of chromaffin cells which were able to migrate to the adrenal medulla. Moreover, the dependency of adrenal medulla formation on SCPs migration along nerves was elegantly demonstrated by achieving specific ablation of preganglionic motor neurons, resulting again in a strong (78%) reduction of chromaffin cells, with the remaining chromaffin cells presumably derived from earlier neural crest migrating cells. Another key finding from this study is the demonstration of an early lineage segregation of sympathoblasts and chromaffin cells, which were until recently considered to originate from a common sympathoadrenal progenitor (133, 134).

### Stem Cells and Regenerative Medicine in the Adrenal Medulla

The question of whether stem/progenitor cells with regenerating abilities persist in the post-natal adrenal medulla has not been extensively investigated. Initial *in vitro* studies showed that cells with progenitor characteristics could be enriched from bovine (135) and human (136) medullary extracts; these cells could also generate spheres expressing progenitor cell markers such as Nestin (a type IV intermediate filament protein expressed in multipotent neural stem cells), CD133, and Notch1. Subsequently, by using a Nestin–GFP transgenic mouse model, it was shown that Nestin^+^ cells (accounting 6% of medullary cells) were negative for both Tyrosine-hydroxylase and chromogranin A (two markers of differentiated chromaffin cells), suggesting that Nestin was not expressed by mature chromaffin cells. Isolated Nestin-GFP cells were also able to generate spheres, which were able to differentiate into chromaffin cells and neurons. This was also confirmed *in vivo* where mice were subjected to repeated immobilization stress; again, the progeny of Nestin^+^ cells, investigated using an inducible nestin–Cre mouse line, was found to include cells with glial, neuronal, and chromaffin identity (137). Chromaffin-like cells have been recently derived from hESCs via a multistep protocol involving first differentiation toward neuroectoderm-like caudal neural progenitors via TGFβ and GSK3β inhibition followed by establishment of neural crest stem/progenitor cells neurospheres in the presence of Fgf2 and Bmp2. Further treatment of these neurospheres with Bmp4 or with dexamethasone plus phorbol 12-myristate 13-acetate (PMA) induced a strong up-regulation of markers of mature chromaffin cells, such as tyrosine hydroxylase and Phenylethanolamine N-methyltransferase (138). The generation and culture of functional chromaffin-like cells could be employed in the field of regenerative medicine, specifically in cases of neuroendocrine/neurodegenerative diseases, and also for pain management.

## Thyroid Gland

### Endocrine Function in the Thyroid Gland

The thyroid is a butterfly-shaped gland located in front of the trachea. Its main function is to regulate body metabolism by producing thyroid hormones T4 and T3 from iodine. Thyroid tissue is composed by two cell types: follicular cells, responsible of thyroid hormones secretion, and parafollicular cells (or C cells), which secrete the hormone calcitonin, involved in calcium regulation. The thyroid gland is controlled by the pituitary gland through secretion of TSH which stimulates the thyroid gland to produce more hormones.

### Key Pathways Guiding Thyroid Gland Development

**Figure 5:**
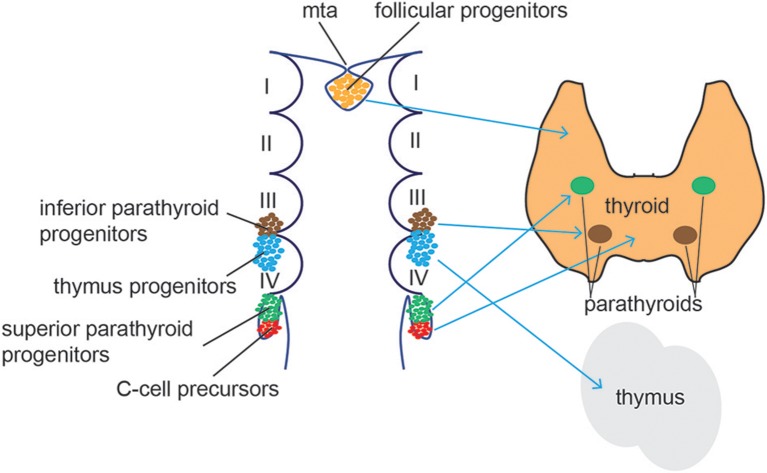
Thyroid/Parathyroid development. Follicular thyroid progenitor cells (orange) derive from the midline thyroid anlage (mta), an endodermal tissue in the floor of the pharynx just caudal to the 1st pharyngeal arch. The superior parathyroid glands (green) originate from the 4th brachial pouch while the inferior parathyroid (blue) and the thymus develop from the 3rd brachial pouch. C cells (red) differentiate from the ultimobrachial body, below the 4th brachial pouch.

Follicular cells arise from the thyroid anlage, a group of foregut endodermal cells located on the midline of the posterior mouth cavity, while parafollicular cells differentiate from the ultimobranchial bodies, a structure derived from the fourth pharyngeal pouch in the developing neck (Figure 5). Recent reports demonstrate that parafollicular C cells develop from pharyngeal endoderm and not neural crest cells, as previously suggested (139, 140). Using a dual mouse lineage tracing strategy, Johansson and collaborators provided direct evidence that C cells derive from Sox17-expressing endodermal progenitors and not from Wnt1-expressing neural crest-derived progenitor cells (141). Both cell types migrate from their original sites to form the definitive thyroid gland (142, 143). During this process, thyroid anlage cells bud by proliferation and invade the surrounding mesenchyme. The thyroid primordium bifurcates bilaterally and migrates toward the larynx and proximal trachea, a process accompanied by intense thyroid progenitor proliferation. Once the left and right thyroid lobes are formed, functional cellular differentiation takes place, which in humans occur after the eleventh week of gestation (144).

During early specification of thyroid cells, exclusive cooperation of the transcription factors Haematopoietically-Expressed Homeobox Protein (Hhex), NK2 Homeobox 1 (Nkx2-1, also known as thyroid transcription factor, Ttf1), Pax8 and Forkhead Box E1 (Foxe1, also known as thyroid transcription factor 2, Ttf2) have been shown to be essential (145, 146). Indeed, genetic deletion of these transcription factors individually resulted in severe thyroid hypoplasia or lack of thyroid formation (147–151). Hhex plays a role in maintaining total progenitor cell numbers in the budding epithelium, while it is not required for thyroid precursor specification (145). Nkx2-1 is not essential for initial specification of the thyroid gland, but is required for the development and morphogenesis. Nkx2-1^−/−^ mice develops a thyroid rudiment which degenerates through apoptosis (152), highlighting its primordial role in pharyngeal endoderm-derived tissues. Pax8 acts as a regulator of thyroid precursor survival. Similar to Nkx2-1^−/−^ mice, Pax8^−/−^ animals show a progressive degeneration of thyroidal primordium (145). Finally, Foxe1 plays a role in migration of thyroid precursor cells. Although the thyroid primordium is formed, progenitor cells in Foxe1-null animals remain attached to the pharyngeal floor whereas in wild-type embryos they are detached from the pharynx cavity and begin to migrate (149). Altogether, the coordinated expression and regulation of these key transcription factors in a timely manner drives the generation, expansion and migration of progenitor cells to form the definitive thyroid tissue.

### Stem Cells and Self-Renewal in the Thyroid Gland

The first indirect evidence that the thyroid gland was an organ endowed with extremely slow self-renewal potential was provided in 1974 through observation of follicular cell proliferation (153). Subsequently, cell population kinetics was studied *in vivo* in dog thyroids via bromodeoxyuridine incorporation and *in vitro* in human thyroid slices, suggesting a complete turnover of ~8.5–14.4 years (154). These and other studies in the 1980s led to the hypothesis of the existence of an unknown number of resident adult stem cells in the thyroid governing this slow self-renewal process (155). Initial efforts to isolate thyroid stem cells in mice showed the existence of a small pool of cells expressing the stem cell markers Oct4, nucleostemin and the ATP binding cassette (ABC)-dependent transporter ABCG2 (the latter endowing cells with the ability to efflux the vital dye Hoechst 33342, also referred to as side population), while expressing low levels of differentiated thyroid markers such as thyroglobulin, TSH receptor, thyroid peroxidase, Pax8, or thyroid transcription factor 1 (Titf1). The same authors also demonstrated that ABCG2-expressing cells were located in the interfollicular space of the thyroid gland but not in cells lining the follicles (156).

In humans, another evidence of the existence of thyroid stem cells were reported by Thomas et al. (157); histologic staining and cultured cells derived from goiters (abnormal enlargement of the thyroid gland), showed a subset of cells expressing the stem cell marker Oct4, and the early endodermal markers Gata4 and Hepatocyte Nuclear Factor 4α (HNF4α) markers while they were negative for the differentiated cell marker thyroglobulin. Interestingly, these markers were found not to be expressed in the differentiated rat thyroid cell line FRTL5 cell line, while they were expressed in undifferentiated thyroid carcinoma cell lines.

Lan et al. isolated adult thyroid stem cells as side population from human goiters by employing Hoechst 33342 staining (expression of ABCG2) followed by fluorescence-activated cell sorting (FACS). Those cells, which accounted for 0.1% of the total cell population, showed stem cell morphological characteristics (smaller in size and higher nucleus to cytoplasm ratio compared with differentiated cells) and expression profiles compatible with an undifferentiated population, and were able to differentiate *in vitro* into thyroid cells upon TSH treatment. Interestingly, spheres established from dissociated thyroids were able to grow *in vitro* in a medium enriched with Egf and bFgf but without TSH, and contained a 50-fold enrichment of side population cells. When stem cells isolated from these 3D structure (named thyrospheres) were grown as monolayer or embedded in collagen, differentiation under the influence of TSH was observed (expression of Pax8, TG, NIS, TSHr, and TPO as well as 125 iodide uptake in response to TSH). These data proved the ability of adult human goiters-derived thyrospheres to differentiate into functional thyroid cells (158). In 2008, Fierabracci and collaborators generated spheroids in culture from human healthy thyroids; their thyrospheres could self-replicate *in vitro* and generate thyroid hormones upon differentiation conditions (159).

Fgfs and Bmps are essential signaling pathways for thyroid cell fate induction. Revest and collaborators reported lack of thyroid glands in Fgfr2b-deficient mice (160). The same phenotype was reported on Fgf10 knockout mice (161), suggesting that Fgf10 could act as a Fgfr2b ligand during thyroid development. Other Fgfs, like Fgf2 and Fgf8, have been involved in thyroid development (162). *In vitro* studies using mouse embryonic stem cells supports the evidence of FGF signaling in differentiating thyroid cells. Longmire and collaborators showed that Fgf2 and Bmp4 are required to generate functional thyroid cells from human and mouse ESCs/ iPSCs (163), reinforcing the notion that these signaling pathways are important during development of the thyroid glands.

### Stem Cells and Regenerative Medicine in the Thyroid Gland

Studies in thyroid regeneration after partial thyroidectomy (PTx) showed that the central areas of both lobes act as the proliferative centers (164). Microarray analysis performed after PTx reveal increased expression of embryonic development pathways, suggesting potential dedifferentiation events or activation of resident stem/progenitor cells. Interestingly, levels of serum T4 hormone, which were decreased after PTx, recover to normal after a week. Accordingly, increases in TSH were detected after PTx to stimulate the gland to produce more T4. In fact, TSH is known to play a role in promoting undifferentiated progenitor/stem cells to transform into mature thyroid follicular cells (158, 165).

Zhang and collaborators have postulated a model for the origin of thyroid carcinoma from adult progenitor cells based on their cell of origin and the levels of differentiation (166), however the low turnover of thyroid gland cells make it difficult to study the relationship between normal and thyroid cancer stem cells.

Several groups have generated thyroid progenitor and mature functional thyroid cells from both mouse and human pluripotent stem cells (163, 165, 167–169). Pioneering work by Arufe and collaborators showed the ability of mouse ES cells to differentiate toward thyroid follicular cells when cultured in serum-free medium supplemented with TSH (165). In 2012, evidence of *in vivo* functionality was demonstrated using mouse ES-derived three-dimensional thyroid follicular cells. Differentiated cells, obtained through transient overexpression of the transcription factors Nkx2-1 and Pax8, were able to restore thyroid hormone plasma levels once implanted into athyroid mice (170).

Modulation of Tgfβ, Bmp and Fgf signaling pathways lead to the generation of primordial thyroid progenitor cells from mESCs (163), that could be further matured to functional, transgene-free thyroid follicular organoids able to secrete thyroid hormones and rescue hypothyroid mice after transplantation (168). Interestingly, iPSCs-derived human thyroid progenitor cells were obtained from healthy donors and patients with hypothyroidism (168). More recently, functional iPSCs-derived human thyroid follicular cells showed the ability to express thyroid proteins and secrete thyroxine *in vitro* (169).

## Parathyroid Glands

### Endocrine Function in the Parathyroid Glands

The parathyroid glands are four small glands that produce and secrete parathyroid hormone (PTH) into the bloodstream. Located behind the thyroid gland, parathyroid glands control bodily calcium levels, playing a crucial role in regulating nervous and muscular systems, bone calcium release and calcium reabsorption in the kidney.

### Key Pathways Guiding Parathyroid Glands Development

The parathyroids are endoderm-derived tissues that form from the third and fourth pharyngeal pouches in humans (171), before migrating to the ventral midline of the pharyngeal and upper thoracic region (Figure 5). Studies in mice demonstrated a common origin of parathyroid and thymus cells in early organogenesis. The parathyroid-thymus primordia separate around e12.5 in mice during the ventral migration, a process mediated by cell adhesion molecules and Bmp4 signaling (172, 173). Expression of the transcription factor glial cells missing 2 (Gcm2) is essential for parathyroid specification. Gcm2^−/−^ mice lack parathyroid glands and develop primary hypoparathyroidism (174) and human Gcm2 mutations have been associated with dysregulated parathyroid hormonal levels (175, 176). Gcm2 expression and patterning in the developing parathyroid gland is tightly controlled by Shh signaling (177, 178). Shh controls the expression of the transcription factors Tbx1 and Gata3 that, together with Gcm2, restrict the parathyroid cell fate of the third pharyngeal pouch (179). Indeed, Shh^−/−^ mice showed smaller, aparathyroid primordia, due to the inability to activate Gcm2 expression. Moreover, Shh was found to be active in both dorsal endoderm and the adjacent neural-crest derived mesenchyme. Bain and collaborators showed evidence that Shh signals from both tissues promote parathyroid specification and organogenesis (180).

### Stem Cells in the Parathyroid Glands

Resident adult stem cells in the parathyroids have been poorly characterized. Human parathyroid-derived stem cells (hPDSCs) were isolated from surgically removed parathyroid glands via enzymatic digestion (181). *In vitro*, selected clones of hPDSCs showed characteristic of adult stem cells as they: (i) could differentiate toward osteogenic, chondrogenic and adipogenic lineages using appropriate induction media, (ii) were positive for mesenchymal stem cell markers and negative for hematopoietic and endothelial markers, (iii) and showed telomerase activity and self-renewal capacity.

Hyperparathyroidism usually occurs due to clonal parathyroid hyperplasia or adenomas of the gland (182, 183). Parathyroid tissue from 20 patients with hyperparathyroidism showed clonal cellular expansion of resident stem cells in both malignant and benign parathyroid tumors, assessed by immunohistochemistry and FAC-sorting for the tumorigenic stem cell makers CD44/CD24 (184). The authors suggested the involvement of a population with stem cell markers in the development of parathyroid hyperplasia.

### Stem Cells and Regenerative Medicine in the Parathyroid Glands

Differentiation of parathyroid-like cells from pluripotent stem cells has been achieved *in vitro* using mESCs. Bingham et al. reported the generation of parathyroid hormone (PTH)-secreting cells expressing both intermediate endoderm progenitor markers (Cxcr4, Eya1, Six1, and Pax2) and parathyroid-specific markers (glial cell missing-2 [Gcm2], CCL21, calcium sensing receptor [CaSR], and PTH) (185).

## Gonads

### Shared Developmental Stages of the Gonads

**Figure 6:**
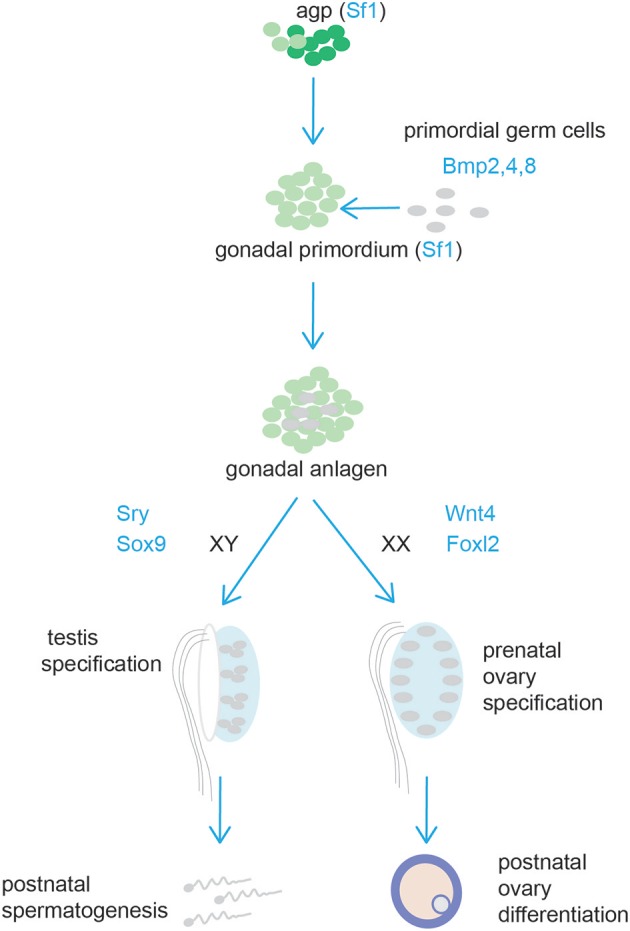
Schematic representation of testis and ovary development. Cells from the adrenogonadal primordium (agp) form the gonadal anlagen. The gonadal anlagen is invaded by migrating primordial gem cells that derive from the region of the forming hindgut. Expression of Sry/Sox9, and Wnt4/Foxl2 determine gonad differentiation into testis or ovaries, respectively.

The gonads and the adrenal cortex originate from the agp (see above, adrenal cortex section). Gonadal primordia develop as paired thickenings of the coelomic epithelium known as the urogenital ridge (Figure 6). Initially, the mammalian gonads develop identically in both female and male embryos. The early mammalian gonad is in fact an undifferentiated primordium composed of bipotential precursor cells that can follow one of two possible fates to become either a testis or an ovary. In mice, development of the urogenital ridge starts at around e11 and continues until e11.5-12.0 when sexual differentiation begins. Primordial germ cells (PGCs) (the precursors of oocytes and spermatozoa in the ovaries and testes, respectively) do not arise within the ridge but migrate from an entirely separate source; at around e7, PGCs are seen in mice in the region of the forming hindgut. The appearance of PGCs is concomitant with increase in the activity of Bmp2, Bmp4, and Bmp8. Early studies showed that ablation of Bmp4 (186) and Bmp2 (187) in mouse embryo resulted in lack and severe reduction of PGCs number, respectively.

Between around e9.0 and e11.5, PGCs migrate to the genital ridge (188, 189). During migration and after settling in the gonad the PGCs divide mitotically, and their number increases rapidly. By e13.5 the genital ridge contains thousands PGCs from an initial population of 10–100 in mice (190).

## Testis

### Key Pathways Guiding Testis Development

In the male gonad, PGCs give rise to T1-prospermatogonia and enter G0 mitotic arrest, a state in which they remain until after birth (191). T1-prospermatogonia resume proliferation during the first week after birth when they become T2-prospermatogonia and migrate to the seminiferous tubules' basement membrane. These cells give rise to the first round of spermatogenesis concomitant with the establishment of the initial pool of Spermatogonial Stem Cells (SSCs) that maintain spermatogenesis throughout post-pubertal life (192).

Differentiation of testis is marked by polarization of gonadal somatic Sertoli cells that form epithelial aggregates around germ cells. This process leads to the reorganization of the gonad into two compartments: the tubular testis cords (referred to as seminiferous tubules after birth), which consist of Sertoli cells and germ line cells, and the interstitial space between the cords, which contains Leydig cells (producing testosterone under the action of LH) and vasculature. Peritubular myoid cells surround Sertoli cells and deposit a basal lamina at the periphery of the tubular structures (193). Post-natally, Sertoli cells form tight junctions with each other that compartmentalize the seminiferous epithelium into basal and adluminal compartments.

In mammals, testis differentiation depends on gonadal expression of the Y-linked gene *Sry*, a transcription factor initiating Sertoli cell differentiation. By e11 in mice and 41–44 days post-ovulation in humans, *Sry* is detected specifically in Sertoli cells (194). Its expression is restricted between e11 and e13 in mice, whilst it is maintained at low levels during gestation in humans. Interestingly, testis-cord formation occurs between e12.5 and e13, a little later than when *Sry* is detected. Differentiation of testis seems dependent on a critical threshold of Sry-expressing cells. About 10% of Sertoli cells were found not express Sry in testes of chimeric XX↔XY embryos, while gonads composed of <30% Sry-expressing cells developed as ovaries (195). Experiments with transgenic mice demonstrated that the early male marker Sox9 is up-regulated by the transient expression of Sry specifically in Sertoli cell precursors (196). More recently, this was confirmed by the generation of a mouse antibody against Sry (197). Further studies revealed that Sry binds to multiple elements within Sox9 enhancer in mice and it does so along with Sf1 (198). The activation of a network of genes downstream of Sox9 then promotes male development while simultaneously blocking the genes that drive ovarian development [reviewed by (199)].

The fact the Sf1 is essential for gonadal development is widely accepted (200). During the early phase of proliferation (e11.5-12.0) Sertoli cells (and interstitial cells) derive from the division of cells expressing Sf1 of the coelomic epithelium. Sf1 is subsequently downregulated (or completely lost) in the coelomic epithelium and proliferation continue in Sf1^−^ cells at and below the coelomic epithelium to produce only interstitial cells (201).

The importance of Sry in testis development is highlighted by numerous mutations causing sexual-development disorders (202), yet little is known about its regulation. Nevertheless, three key transcription factors, Gata4, Friend of Gata protein 2 (Fog2) and Wt1, have been implicated in the transcriptional or post-transcriptional regulation of the gene [reviewed by (202)].

### Stem Cells and Self-Renewal in Testis

**Figure 7:**
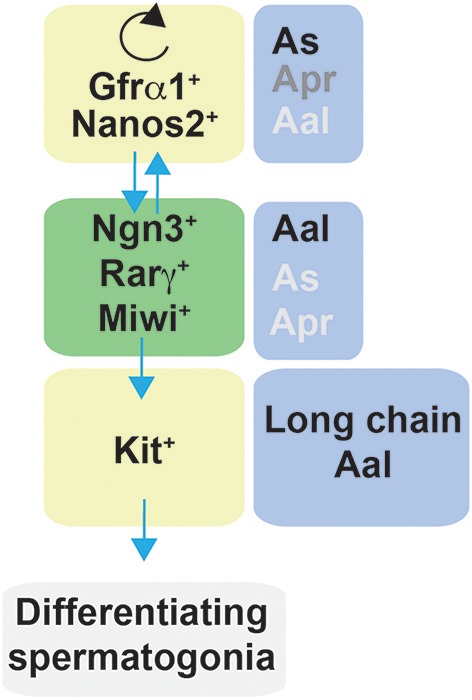
Schematic representation of factors affecting self-renewal and differentiation of spermatogonia in mice. Spermatogonia are classified as Asingle (As), Apaired (Apr), and Aaligned (Aal) according to the number of cells contained in a syncytium. In steady-state, a subset of Gfrα1/Nanos2 expressing cells function as stem cells with the ability to self-renew their population. Gfrα1^+^ spermatogonia have the ability to generate cells that lose the expression of Gfrα1 and become Ngn3^+^/Rarγ^+^/Miwi2^+^, which can retain the stem cell potential but mostly become Kit^+^ cells, and therefore are committed to terminal differentiation. In regenerative contexts, Ngn3^+^/Rarγ^+^/Miwi2^+^ can regain Gfrα1 expression contributing to the self-renewing pool.

Spermatogenesis, the process that throughout the life of males produces sperm, represents a typical example of a supported stem cell system. Spermatogenesis occurs in the seminiferous tubules where spermatogonia that reside on the basement membrane undergo self-renewal divisions and proliferate to form spermatogonial clusters. In rodents, three types of spermatogonia have been identified, namely Type A, intermediate and B. Type A cells are the most undifferentiated and have been classified by morphological analysis into Asingle (As, isolated cells), Apaired (Apr, chain of two connected cells), or Aaligned (Aal, chain of 4, 8 or 16 or more cells), that remain connected by intercellular bridges due to incomplete cytokinesis (Figure 7). According to the prevailing theory in the field, known as the ‘As model', spermatogonial stem cells (SSCs) are As cells (roughly 0.03% of the total number of spermatogonia) that divide into two daughter Apr spermatogonia which further divide into Aal spermatogonia (203, 204). Aal spermatogonia are the source of primary spermatocytes that will enter meiosis and further develop into haploid spermatids and sperm (205). Nevertheless, further studies have revealed that morphology alone is not sufficient to characterize spermatogonial cells. Undifferentiated spermatogonia were firstly identified as being negative for the surface receptor Kit (206, 207). However, more recent studies have revealed a more heterogeneous characterization of undifferentiated spermatogonia and several markers can now be used to identify SSCs. Comparison of gene expression by whole-mount double-staining of seminiferous tubules revealed that the transcription factor Plzf (promyelocytic leukemia zinc-finger) (208, 209) and the calcium dependent cell-cell adhesion glycoprotein E-Cadherin (210) have identical expression patterns and are present in all A spermatogonia (211). In contrast, the cell surface receptor Gfrα1 and the transcription factor Nng3 showed a more heterogeneous expression, where As, Apr and Aal can be stratified into Gfrα1 single-positive, Gfrα1/Ngn3 double positive, and Ngn3 single-positive. The shorter chains of cells have a greater probability of being Gfrα1 single-positive while longer chains tend to be Ngn3 single-positive (211–213). Moreover, the m-RNA binding protein Nanos C2hc-Type Zinc Finger 2 (Nanos2) promotes the male fate while suppressing meiosis in embryonic XY germ cells (214). Recently, pigs with heterozygous and homozygous mutations in Nanos2 were generated using the CRISPR/Cas9 system. Males pigs had an impaired development of testis, specifically homozygous Nanos2 knockout had no germ cells in the presence of intact seminiferous tubules (215). Nanos2 was found to be almost exclusively expressed in As to Apr cells, whereas Nanos3 is detectable in most undifferentiated spermatogonia (As to Aal) (214). This heterogeneity of gene expression has suggested functional heterogeneity within the same cluster of cells (i.e., As, Apr, Aal) (Figure 7).

Lineage tracing, live imaging and pulse labeling studies have revealed that differentiation of spermatogonia is more complex that previously described and have led to a revision of the traditional “As model.” In steady-state, a subset of Gfrα1^+^ cells resides on the top of the hierarchy (211) and function as stem cells with the ability to self-renew their population while maintaining a constant number of Gfrα1^+^ spermatogonia (216). Moreover, Gfrα1^+^ spermatogonia were shown to continually interchanged between As, Apr and Aal spermatogonia through a combination of incomplete division and syncytial fragmentation. At the same time, all categories of As, Apr, and Aal Gfrα1^+^ spermatogonia had the ability to generate cells that lose the expression of Gfrα1 and become positive for Ngn3. Ngn3^+^ cells, independently from the chain length (including As cells), are destined for differentiation and become Kit^+^ which undergo several further rounds of cell division and are committed to terminal differentiation. Eventually all Kit^+^ cells are derived from Ngn3^+^ cells (211, 213). Interestingly, these studies also demonstrated that Ngn3^+^ cells retain the capability of regaining GFRα1 expression, fragmenting into single cells or shorter syncytia (through breaking of intercellular bridges), and contributing to the long-term stem cell pool. This reversion is rare in homeostasis but becomes more frequent during regeneration, for example after tissue insult by a cytotoxic reagent or transplantation (211, 217). Therefore, Ngn3^+^ spermatogonia have been referred to as “potential stem cells” (211, 217). In this context, further studies have identified other subpopulations of undifferentiated cells that contribute to the self-renewing pool. Carrieri et al. identified a novel population of Ngn3^+^ spermatogonia that express the Piwi protein Miwi2, which was shown by cell ablation to be crucial for efficient regenerative spermatogenesis after injury (218). More recent studies have further characterized germline stem cells; for example, Gfrα1^+^ cells comprise subpopulations that express the transcription factor Pancreatic and duodenal homeobox 1 (Pdx1) (219), the Erb-B2 Receptor Tyrosine Kinase 3 (Erbb3) (220), Inhibitor of differentiation 4 (Id4) (221) and Shisa family member 6 |(Shisa6) (222).

Spermatogonia reside within specialized microenvironments - referred as “niches” - in the basal compartment of seminiferous tubules. A undifferentiated spermatogonia, including Gfrα1^+^ subpopulation, localize preferentially to the area adjacent to the vasculature network of arterioles and venules that accompanies interstitial cells (216, 223, 224). On making the transition into differentiating spermatogonia, they migrate out of these areas and disperse over the entire basal compartment of the seminiferous epithelium (225). Interestingly, live-imaging revealed that Gfrα1^+^ cells intersperse between Ngn3^+^ and Kit^+^ spermatogonia and are in constant movement in the basal compartment where they actively migrate between Sertoli cells (216). Such a microenvironment can be designated as an open stem cell niche.

Although the molecular mechanisms governing the maintenance and fate of A undifferentiated spermatogonia are yet not fully understood, Sertoli cells are widely regarded as key contributors to the maintenance and differentiation of SSCs, being the main source of the Glial -derived neurotrophic factor (Gdnf) (the ligand for GFRα1 receptor complex), and Fgf2 (226). For example, *in vivo* overexpression and loss-of-function models show that the dosage of Gdnf regulates accumulation/depletion of undifferentiated spermatogonia (227), and *in vitro* stimulation with Ggnf leads to proliferation of GFRα1^+^ cells (212). Gdnf-mediated proliferation of SSCs involves regulation of Src family kinases, Yes, Lyn and Fyn. Gdnf activates Src family kinases, which further stimulate the phosphoinositide 3-kinase (PI3K)/Akt pathway (228) and up-regulates N-Myc expression to promote SSCs proliferation (229). More recently, further studies revealed that Gdnf production is regulated by the canonical Notch pathway (191, 230) via the transcriptional repressors Hes1 and Hey (231). Fgf2 was shown to expand GFRα1^+^ cells, although these cells had a distinct phenotype from Ggnf. Fgf2 expanded a retinoic acid receptor γ (Rarγ) expressing subset of cells showing Fgf2 function to be more appropriate for spermatogonial differentiation (226). It is known that retinoic acid (RA), which is synthesized from Vitamin A, is required for spermatogonial differentiation (232, 233). The generation of Kit^+^ spermatogonia was blocked in the testes of Vitamin A deficient mice and reinitiated after administration of Vitamin A. Lineage-tracing analysis revealed that Ngn3^+^ cells (but not Gfrα1^+^), which specifically express Rarγ, transit to Kit^+^ cells rapidly and efficiently in response to RA (234). Fgf2 signaling is dependent on Map2k1 pathway activation to drive SSC self-renewal via upregulation of the transcription factor Ets variant 5 (Etv5) and transcriptional repressor B-cell CLL/lymphoma 6, member B (Bcl6b) (235). Another study indicated that Fgf2 may regulate SSCs proliferation *in vitro* via phosphorylation on Akt and Erk1/2 pathway (236). Finally, it should be mentioned that the activation of the Wnt/β-catenin pathway is thought to drive the transition from Gfrα1^+^ to Ngn3^+^ spermatogonia, and signaling is likely initiated by Wnt6, which is uniquely expressed by Sertoli cells (222, 237, 238). Evidence for the importance of Sertoli cells as supporting/regulatory cells also comes from *in vivo* knockout experiments, which identified Sertoli cell specific genes, for example Connexin 43 (cx43), Swi-independent 3a (Sin3a), cytochrome P450 enzymes (Cyp26b1), and Ets related molecule (Erm), some of which play a role in the above pathways, that are essential in supporting germ cell proliferation and/or survival (239–242) and normal spermatogenesis (243).

Another factor which is important for the maintenance of the SSCs pool is oxygen availability. The microenvironment where SSCs reside can be described as being low in oxygen (or hypoxic), a condition that induces the activation of transcription factor hypoxia inducible factor 1α (HIF1α) and can inhibit cell differentiation (244). Staining of adult testis revealed the expression of HIF1α in the stem cell niche along the basement membrane of the seminiferous tubules, while the signal diminishes as cells differentiate, implying a possible role of Hif 1α in germ cell development (245).

Another important question is the extent to which the knowledge acquired using rodents can be applied to humans. Human spermatogonia are characterized by their nuclear morphology and staining with haematoxylin as Adark and Apale spermatogonia (246). Adark spermatogonia are thought to function as reserve stem cells, whilst Apale spermatogonia are progenitors of spermatocytes. Nevertheless, their identity, self-renewal and differentiation abilities are just beginning to emerge. Prepubertal human spermatogonia showed expression of genes important in mouse SSCs regulation (247). Immunohistochemistry on tubule sections revealed human spermatogonial cells share some (i.e., GFRα1) of the markers found in rodents (248). More recently, three independent groups revealed using single-cell RNA-sequencing in human testis clear evidence for heterogeneity and identified distinct cell clusters including SSCs (249–251). These findings provide a starting point for further studies, such as the evaluation of SSC frequency and assessment of SSC activity (252).

Leydig cells, the testosterone-producing cells of the adult testis, derive from stem Leydig cells, spindle-shaped cells that lack steroidogenic cell markers (253). Once formed, Leydig cells rarely die or divide. Nevertheless, their depletion in conditions such as ethane dimethanesulfonate is followed by the appearance of new, fully functional adult Leydig cells (254, 255), which are thought to arise from precursors stem cells (254). Very recently, it was shown in male rats that Fgf-homologous factor-1 (Fhf1 or Fgf12), an intracellular protein, is abundant in Leydig cells and that injection of Fhf1 resulted in Leydig cells regeneration from precursor stem cells in rats where Leydig cells were pharmacologically ablated (256).

In contrast, one study reported that complete ablation of Sertoli cells *in vivo*, either in fetal life (e16.5) or post-natal life, did not lead to repopulation of the testis with new Sertoli cells, indicating Sertoli cells do not possess regenerative capacity and no stem Sertoli cells are present in adult testis (257).

### Stem Cells and Regenerative Medicine in the Testis

In recent years, the pluripotency characteristics of SSCs has emerged. For example, the generation of pluripotent embryonic stem like cells was established from neonatal mice testis (258). Similarly, in humans SSCs yielded human testis-derived embryonic stem-like cells (htESLCs) (259, 260); htESLCs were shown to differentiate *in vitro* into derivatives of all three germ layers including neural, epithelial, osteogenic, myogenic, adipocyte, and pancreatic lineages (261). Therefore, SSCs are considered a feasible source for applications in regenerative medicine.

Adverse effect of cancer treatments in men include long-term infertility. If cancer occurs after puberty sperm cryopreservation is the simplest and the most effective method to preserve fertility, nevertheless in prepubertal patients this is not an option. The self-renewal and differentiation abilities of SSCs make these cells a promising tool in the treatment of infertility. To this end, cryopreservation of testicular tissue before chemo-therapy and later autotransplantation of SSCs could theoretically be used to restore fertility. In this context promising results have been obtained in animals. Already in 1994, Brinster and Zimmermann showed that male mice stem cells injected into seminiferoustubules repopulated sterile testes and donor recipients produced mature spermatozoa (262). Human germ cells xenotransplanted to testes of busulfan-treated mouse (with suppressed spermatogenesis) testes survived for at least 6 months and proliferated during the first month after transplantation, however no human-differentiating spermatogonia were identified (263). Similarly, spermatogonia in the testis of a prepubertal boy were shown to migrate to the basement membrane of the mouse recipient seminiferous tubule and were maintained as germ cells (247). Human testicular cells from adult men were isolated, maintained and proliferated *in vitro* for longer than 20 weeks. In 4 out of 6 men, even after prolonged *in vitro* culture, xenotransplantation to mice demonstrated the presence of functional SCCs (264). Importantly, testicular cells from a 6.5- and 8-year-old boys were cultured *in vitro* for at least 15.5 weeks (265). Elhija et al. established a 3D agar culture system which was able to induce germ testicular cells from mice to generate morphologically normal spermatozoa (266). Sato et al. reported the use of an *in vitro* organ culture method that supported complete mouse spermatogenesis (267, 268); subsequently this methodology was used to generate viable sperm, which through micro-insemination resulted in healthy offspring (269). Although it might take a while before the first clinical trial of SSCs autotransplantation is granted, these pre-clinical data are promising.

## Ovary

### Key Pathways Guiding Ovary Development

**Figure 8:**
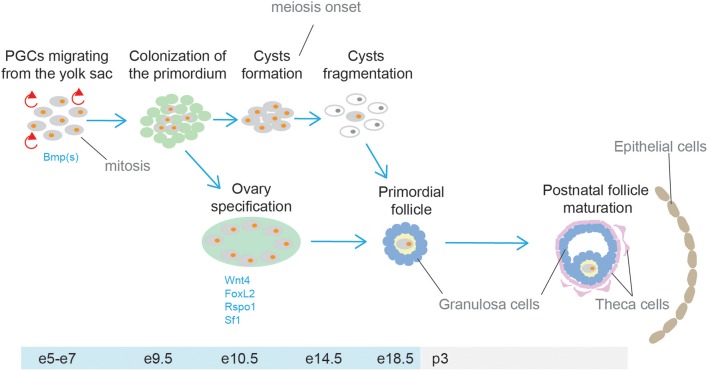
Schematic representation of ovary development. Primordial germ cells (PGCs) colonize the gonadal primordium and undergo mitotic division with incomplete cytokinesis producing cysts. Subsequently, germ cell cysts undergo breakdown to produce primordial follicles, consisting of a single oocyte surrounded by pre-granulosa cells. During sexual maturation, primordial follicles develop further eventually becoming potential fertilizable eggs at sexual maturity.

In females, PGCs divide by mitosis with incomplete cytokinesis until around e13.5 in mice and 11–12 weeks in humans producing germ cell cysts (also called germ cell nests) (Figure 8) (270). Mitotic division ends and germ cells enter meiosis-I and arrest in the diplotene stage of prophase-I eventually becoming oocytes (271). Germ cell cysts start undergoing breakdown (starting at around e18 until post-natal day 5) to produce primordial follicles (primordial follicle pool) consisting of a single oocyte surrounded by pre-granulosa cells (272–274). At this time, the ovary is reorganized into morphological compartments, the cortex (containing primordial follicles) and the medulla. During a process called folliculogenesis primordial follicles further develop to become potential fertilizable eggs at sexual maturity. During a first phase (the preantral phase), primordial follicles mature into primary and secondary follicles. In a second phase (the antral or gonadotropin-dependent phase) granulosa cells secrete follicular fluid generating fluid-filled antral follicles. After the onset of puberty, activation and further maturation of follicles lead to oocytes ovulation. Just before ovulation, oocytes complete the first meiotic division and begin the second meiotic division which is completed only after fertilization.

Factors that determine ovarian specification include members of the Wnt/β-catenin pathway. Expression of Wnt4 is firstly detected from e10 onwards. When sex specific differentiation begins, Wnt4 is downregulated in males and continues to be expressed in females. Ovaries of Wnt4^−/−^ appeared masculinized (absence of Müllerian duct and development of Wolffian duct) indicating that Wnt4 is a determinant of the female gonad (275). A mutation in the human R-spondin1 (*RSPO1*) gene, was shown to be responsible for female-to-male sex reversal. Moreover, the same study reported that Rspo1 is expressed specifically in XX gonads of mice during the critical stage (e13–15) of gonad differentiation (276). Female *Rspo1*^−/−^ mice showed male phenotypic features similar to what observed in Wnt4^−/−^ mice (277). Another factor involved in ovarian determination is the transcription factor Foxl2, which is detected in female mice as early as e12.5. *Foxl2*^−/−^ female mice revealed Foxl2 is required for ovarian follicle formation (278, 279). Moreover, Wnt4^−/−^Foxl2^−/−^ double knockout ovaries resulted in the formation of testis tubules and harbored well-differentiated spermatogonia (280).

### Stem Cells and Self-Renewal in Ovary

For many years, the mammalian ovary was thought to contain at birth a fixed non-renewing pool of oocyte-containing follicles, that are depleted to exhaustion with aging (281). However, in 2004, Johnson et al. (282) challenged this notion. The group counted the number of atretic (degenerating follicles) and non-atretic (healthy) follicles in ovaries of mice. Based on the number of degenerating follicles at any given time under normal conditions they predicted complete exhaustion of the follicle reserve by young adulthood. Nevertheless, the number of non-atretic follicles declined less than expected. Thus, they speculated that germ line stem cells are present in the post-natal ovary of mice. Not surprisingly, this study ignited a debate on the possibility of post-natal neo-oogenesis in mammals (283, 284), and prompted follow-up investigations. Convincing evidence of the presence of female germline stem cells (FGSCs) [also known as oogonial stem cells (OSCs) (285)] in the mammalian ovary was provided for the first time in 2009 by Zou et al. Firstly, putative FGSCs were identified in neonatal and adult mice ovaries by dual immunofluorescence analysis of BrdU incorporation and mouse vasa homolog (Mvh, a germ-cell marker). Subsequently, FGSCs were isolated from neonatal (nFGSCs) and adult (aFGSCs) mice ovaries by two-step enzymatic digestion and immunomagnetic isolation of Mvh-positive cells. These cells were maintained in culture for months and expressed markers of germline cells and proliferation. Furthermore, when GFP labeled aFGSCs were transplanted into ovaries of infertile mice (sterilized by pre-treatment with cyclophosphamide and busulfan), histological evaluation after 2 months showed that ovaries had many oocytes at all stages of development, including GFP-positive oocytes, suggesting that oocytes can be regenerated in sterile recipient females by transplantation of FGSCs. Ultimately, the transplanted mice produced offsprings that had the GFP transgene (286). Following this study, within a short period of time, similar data were generated. A wealth of literature reported the isolation of mitotically active germ cells from adult animals mainly using magnetic-assisted cell sorting or FACS and subsequent culture of the isolated cells (287–295) [also reviewed by (285)]. Importantly, by the use of FGSCs intragonadal transplantation-base approaches, these studies also confirmed the functional capacity of mouse FGSCs to restore ovarian function and produce offsprings (287, 294, 296).

For a few years, possibly partly due to the lack of appropriate methodology (297), the question of whether FGSCs actually contribute to oocytes during *de novo* folliculogenesis in female adult mice under physiological conditions remained unresolved (298–300). Finally, compelling evidence was provided by the use of a tamoxifen-inducible system that traced Oct4- expressing cells permanently marked with enhanced yellow fluorescent protein (EYFP) in post-natal mouse ovaries. This line of evidence proved the existence of active ovarian germ stem cells *in vivo* and their function in replenishing the primordial follicle pool under physiological conditions (301). Soon after, this result was confirmed by inducible ablation of premeiotic germ cells undergoing differentiation into oocytes driven by the promoter of Stimulated by Retinoic Acid gene 8 (Stra8). With this approach, the study demonstarted that new oocytes are formed in ovaries during adult life and that some of these oocytes contribute directly to the pool of oocytes used for natural reproduction (302).

Aside from the numerous animal studies that have populated the literature since the traditional view of a finite pool of oocytes was challenged (282), human investigations have also emerged. A significant progress in the field was made when viable Mvh^+^ cells were isolated from human ovarian cortical tissue and maintained *in vitro* where they spontaneously generated oocytes as confirmed via morphological and gene expression analyses and attainment of haploid status. Mvh^+^ cells isolated from adult human ovaries were stably transduced with a GFP expression vector, injected in adult human ovarian cortical tissue biopsies and then xenografted into female mice where formation of follicles containing GFP-positive oocytes was observed (303). Similar results were independently obtained by other groups (304–307).

With multiple laboratories now confirming the existence and functional characteristics of FGSCs, new studies have recently emerged in the attempt to investigate their biological activities and regulatory mechanisms (i.e., self-renewal, differentiation, apoptosis). Zhang et al. reported that Cadherin 22 (Cdh22), a member of cadherin family, is required for FGSCs self-renewal via different mechanisms, including interacting with the Jak–Stat and β-catenin signaling pathways (308). In a follow-up study, the same group showed that Cdh22 interacts with Pik3 to phosphorylate Akt3, which enhanced the expression levels of N-Myc and members of the cyclin family to promote self-renewal. Moreover, Gdnf was also shown to be essential for FGSC self-renewal via a more complicated mechanism: Gdnf-Gfrα1 activates Akt3 via PI3K or Src family kinase (Sfk), and Sfk upregulates its target genes, Bcl6b, Etv5, and Lhx1. Nevertheless, Src, the key intermediate factor for SSCs, was not the functional molecule of Sfk family in the Gdnf signal network of FGSCs (309).

The origin of FGSCs has been debated for years. Soon after their first pubblication, Johnson et al. suggested bone marrow as a potential source of female germ cells (310). However, a later study showed, by the use of transplantation and parabiotic mouse models, no evidence that bone marrow cells, or any other normally circulating cells, contribute to the formation of mature ovulated oocytes both in the steady state and after induced ovarian damage (311). A follow-up investigation by Lee at al. reported conflicting conclusions. Transgenic mice with germline-specific expression of GFP underwent bone marrow transplantation (BMT) after injection with busulfan and cyclophosphamide. BMT rescued fertility, but all offspring derived from the recipient germline (312). More recently, positive results came from injection of human bone marrow–derived stem cells (BMDSC) into mice with chemotherapy-induced ovarian damage. BMDSC treatment resulted in production of higher numbers of preovuolatory follicles, metaphase II oocytes, 2-cell embryos, and healthy pups (313).

While much research of stem cells in ovary has focused on FGSCs, indication of normal somatic stem cells has also been provided. The work by Honda et al. showed evidence in newborn mice ovaries of putative thecal stem cells with the ability to self-renew and differentiate *in vivo* and *in vitro*. These putative thecal stem cells formed characteristic anchor-independent round colonies, and, after stimulation, started to differentiate and show characteristic signs of steroidogenesis. Moreover, after transplantation into ovaries these putative thecal stem cells showed aggregation immediately adjacent to developing follicles and in both theca interna and externa during folliculogenesis (314). Using BrdU incorporation and doxycycline inducible histone2B-green fluorescent protein pulse–chase techniques, Szotek et al. identified a putative somatic stem/progenitor cell in the ovarian surface epithelium (OSE) in the adult mouse ovary. Interestingly, Virant-Klun et al. isolated and characterized putative ovarian stem cells obtained from the OSE of the adult human ovary in women with no naturally present oocytes and follicles. Small round cells (2–4 μm) with a bubble-like structure that expressed early embryonic developmental markers were separated and cultured *in vitro* where they proliferated, with some cells reaching a diameter of ~20 μm after 5–7 days (315, 316). Since their discovery, somatic stem cells in the ovary have been of particular interest as these cells may be responsible for ovarian cancer during adult life as well as neo-oogenesis [reviewed by (317)].

### Stem Cells and Regenerative Medicine in the Ovary

The finding of FGSCs in adult human ovaries promts the question whether these cells can be utilized somehow to enhance, prolong or restore fertility in women. Although this might seems a far-fetched scenario, reproductive biologists are already working toward this goal. One possiblity is a procedure known as “Autologous germile mitochondrial energy transfer (AUGMENT),” which involves the use of patient matched FGSCs mitochondria to invigorate oocytes of women with a history of poor egg and embryo quality (318). Another option is based on autologous oocytes transplantation approaches to prolong or restore ovarian function. This would include the development of techniques designed to reconstitute human ovarian tissue which would allow the production of functional eggs from FGSCs entirely *ex vivo* (285). While there is a long way ahead, these techniques would offer women faced with fertility challenges a unique opportunity for bearing a genetically-matched child.

## Endocrine Pancreas

### Endocrine Function in the Pancreas

The pancreas contains both an exocrine and endocrine component, with the endocrine system accounting for ~5–15% of the total pancreas. The exocrine pancreas is composed of acinar cells that secrete digestive enzymes into the pancreatic duct and assist with digestion. The five main cell types of the endocrine pancreas are located within clusters of cells known as the islet of Langerhans, which include: glucagon-producing α-cells, insulin-producing β cells, somatostatin-producing δ cells, ghrelin-producing ε cells and polypeptide-producing PP cells. The α, β, δ, and PP cells play critical roles in maintaining physiologic blood glucose levels, while ε cells play a role during fetal development, but they are virtually absent in the adult pancreas. During periods of elevated blood glucose, which occurs after food ingestion, β cells release insulin which signals to the liver, adipose tissue and skeletal muscle to increase glucose uptake. Conversely, during periods of low blood glucose, α cells secrete glucagon, triggering hepatic glycogen breakdown and glucose secretion into circulation. Somatostatin is known to inhibit both insulin and glucagon secretion, and PP inhibits glucagon release in low-glucose conditions. Together, through the concerted release of these hormones, blood glucose levels are able to remain within a physiologic range (319).

### Key Pathways Guiding Pancreas Development

**Figure 9:**
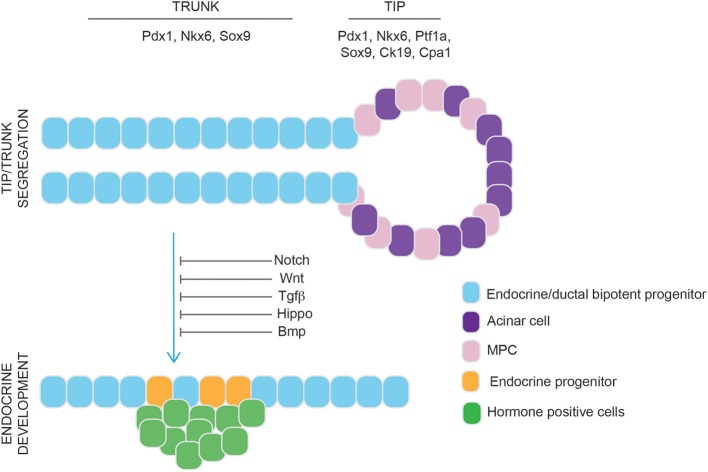
Schematic representation of murine pancreatic development. Multipotential Pancreatic Cells (MPC) (pink) and acinar cells (purple) are located at the tip of the pancreatic epithelium. The trunk contains endocrine/ductal bipotent progenitors (light blue) that migrate out of the epithelium and differentiate to endocrine progenitor cells (orange) which give rise to hormone positive cells (green cells). Endocrine commitment is driven by inhibition of Notch, Wnt, Tgf-β, Hippo, and Bmp signaling pathways.

During embryonic development, the pancreas emerges from the endoderm, a primordial germ cell layer that gives rise to the digestive and respiratory tracts and their derivative organs. Pancreas development begins around e9.5 in the mouse, at which time the dorsal bud emerges from the Pdx1*-*expressing region of the posterior foregut, followed by the ventral bud at e10.0 (320–322). Following a 180-degree rotation around the duodenum, the dorsal and ventral buds fuse to form a single pancreatic anlage. The pancreatic epithelium begins to protrude and undergoes extensive remodeling and formation of a web-like structure, or plexus (320). During this time, the surrounding mesenchyme secretes factors such as Fgf10 and Egf, which are critical for pancreas differentiation and proliferation (320, 323, 324). During plexus remodeling, signaling from the surrounding mesenchyme and polarization of epithelial cells lead to the formation of regions with distinct developmental potential: the tip contains the multipotential pancreatic cells (MPCs) and the trunk contains bi-potent endocrine/ductal progenitors (Figure 9). The multipotent progenitors express Pdx1, Pancreas Associated Transcription Factor 1a (Ptf1a), NK6 Homeobox 1(Nkx6-1), Carboxypeptidase A1 (Cpa), Myc, and Sox9 and provide a source of cells that can become endocrine, ductal and acinar cells (325–332). Cells of the trunk that undergo endocrine and ductal commitment continue to express *Nkx6-1*, a transcription factor required for β cell development, but lose expression of *Ptf1a*, a transcription factor that becomes restricted to acinar cells (333, 334). As cells commit to the endocrine lineage, the pancreatic epithelium and mesenchyme get connected to the vasculature and become less hypoxic, HIF1a (a marker of hypoxia) expression decreases and cells of the epithelium upregulate *Ngn3*, a basic loop helix transcription factor marking all endocrine progenitors (335–337). In order for cells to undergo endocrine differentiation and upregulate Ngn3 expression, Notch signaling must be downregulated (338, 339). In addition to Notch inhibition, recent work by a number of groups have demonstrated that inhibition of Wnt, Tgfβ and Hippo (through the downregulation of its effector Yes Associated Protein, Yap) signaling further enhances human endocrine differentiation (Figure 9) (340–342). The mechanism by which endocrine cells form the islet of Langerhans had been thought to occur as a result of delamination of individual endocrine cells, followed by their subsequent coalescence. This paradigm has recently been challenged by Sharon et al., who proposed that islets form from peninsula-like structures (340, 343–345). In this model, Sharon et al. demonstrated that endocrine cells maintain cellular contact during islet formation: α cells are believed to initially emerge from the trunk region to form the peripheral cells of the islet, followed by the emergence of β cells, which maintain contact with the α cells, in order to form the islet core. At least in the mouse, the final size of the organ is dictated by the number of progenitors that arise during embryonic development and contrary to other organs, such as the liver, the pancreas has very limited proliferative potential in adults (346).

### β Cell Regeneration in the Pancreas

The proliferative capacity of the endocrine pancreas gradually decreases after birth, with β cells showing minimal evidence of proliferation and turnover (347). However, β cell mass increases during pregnancy, suggesting that an increase in β cell mass can occur under physiological conditions (348). Therefore, understanding the mechanisms guiding β cell regeneration has been of particular interest as this knowledge could potentially be leveraged to intentionally increase β cell mass as a treatment for diabetes.

The main mouse models used to study pancreatic regeneration, which have been eloquently reviewed (349), include: pancreatic duct ligation, partial pancreatectomy (removal of 50–75% of the pancreas), chemical induced pancreatitis, and β cell ablation models caused by drug administration, such as alloxan or streptozotocin (349). Partial pancreatectomy (Ppx) has historically been the most common model to study regeneration as it leads to both acinar and islet cell regrowth, making it an interesting model for β cell regeneration (350, 351). Although ductal cells have been identified in some Ppx models to be the source of acinar and β cell regeneration, lineage tracing studies suggest that pancreatic regeneration occurs through self-renewal, where acinar cells generate new acinar cells and β cells generate new β cells (352–355). Using an insulin lineage tracing mouse model to label terminally differentiated β cells, in combination with a Ppx mouse model, Dor et al. identified that the main source of β cell generation is through self-renewal (352). Supporting this idea, using a DNA analog-based lineage tracing method in order to detect each round of cell division, Teta et al. demonstrated that β cells come from pre-existing β cells and not a source of stem/progenitor cells in the adult pancreas (356). Recent publications have shown that β cell heterogeneity exist within the islet, with some β cells having been identified as being more proliferative and immature than other β cells. The heterogeneity that exists could explain the ability of some β cells to be capable of self-renewal, while the less proliferative β cells cannot (357–359).

If, however, β cell regeneration occurs through the proliferation of existing β cells, self-renewal would not explain β cell regeneration in mouse models of type 1 diabetes where near-complete β cell ablation occurs. Interestingly, in a mouse model containing a transgene for an insulin promoter and diphtheria toxin (DT) receptor sequence that can result in up to >99% ablation of β cells following DT treatment, β cell regeneration was shown to occur as early as 15 days post-DT treatment. In this model, using lineage tracing to label glucagon-producing α cells prior to DT-treatment (360), β cell regeneration from α cells was demonstrated. The ability of α cells to transdifferentiate to β cells introduces the idea that endocrine cells retain plasticity, which has been the basis for efforts to identify compounds that could modulate α to β transdifferentiation, but so far with no success (361–363).

Additionally, other studies suggest that insulin expressing cells are in fact the stem cells of the pancreas, being able to generate other exocrine and endocrine tissues (364). More recent work identified pancreatic cells within an islet-depleted cell population, such as ductal tissue, that can generate insulin-expressing cells following transplantation in mice, suggesting a non-endocrine progenitor-like population exists that can also generate insulin producing cells (365).

Overall these studies indicate that the type of stress caused by pancreatic injury and/or the resulting environment may dictate the source of β cell regeneration, thereby adding to the difficulty in deciphering the mechanisms of β cell regeneration in a natural and physiological manner in humans.

### Stem Cells and Regenerative Medicine in Pancreas

In addition to generating β cells through regeneration, using cadaveric donors or human pluripotent-stem cells (hPSCs) offers another source of β cells for therapy. Human cadaveric islets and whole pancreas transplantation have been performed for patients with type 1 diabetes and have demonstrated the ability to normalize glycemia. However, the requirement for numerous donors for each patient, potential requirement for a subsequent transplant, and lack of donors have made hPSC-derived β cells a more compelling source of cells for the treatment of diabetes. The most efficient differentiation protocols to date attempt to recapitulate key stages of pancreas development *in vitro*, including: (1) definitive endoderm formation, (2) posterior foregut patterning, (3) Pdx1 induction, (4) pancreatic progenitor generation (Pdx1^+^/Nkx6-1^+^ cells), (5–6) endocrine commitment (Ngn3^+^ cells), and (7) β-like cells differentiation (Nkx6-1^+^Cpep^+^ cells) (340, 341, 366–370). Pancreatic progenitors offer an appealing source of cells for transplantation as they give rise to all cells of the pancreas following transplantation in mice and can normalize glycemia in an streptozotocin-induced diabetic mouse model of diabetes (367, 368, 371). Supporting the use of hESC for the treatment of diabetes, ViaCyte^TM^ has launched several clinical trials to test the safety of pancreatic progenitor transplantation in humans (NCT02239354, NCT02939118, NCT03162926, NCT03163511). Outcomes of these initial clinical trials will provide knowledge that will be the basis of future hPSC-derived pancreatic transplantations. Although PPs have demonstrated the ability to normalize glycemia in mice, generating β cells *in vitro* from hPSC may allow for a more efficient means to normalize glycemia and contain a more committed endocrine population that would not give rise to other cells of the pancreas, such as acinar cells. Therefore, generating hPSC-derived β cells *in vitro* could provide a cell product that would be more efficient for diabetes therapy. In 2014, two groups identified protocols to generate Nkx6-1^+^/serum-C-peptide (Cpep)^+^ cells from hPSC *in vitro*, and although the hPSC-derived β-like cells could release insulin in response to a glucose challenge, further maturation only occurred following transplantation in mice (372, 373). More recent publications have claimed the generation of more functional β cells from hPSC *in vitro*. However, efficiencies of these published protocols remain poor, with some protocols requiring fluorescence-activated cell sorting using a transgenic INS:GFP reporter cell line, and protocol reproducibility has yet to be confirmed (341, 342, 370). Although signaling pathways guiding human β cell differentiation have been identified in these reports and have helped push the field forward, generating a population of cells that is therapeutically relevant will require extensive improvements in the efficiency, purity, reproducibility, and functionality of hPSC-derived β-like cell directed differentiation protocols.

## Author Contributions

LG and KM contributed to sections on adrenal cortex, adrenal medulla, and gonads. GR-B contributed to sections on thyroid and parathyroids. CG-M, JN, and AG contributed to the section on pituitary. MN and EM contributed to the section on pancreas.

### Conflict of Interest

The authors declare that the research was conducted in the absence of any commercial or financial relationships that could be construed as a potential conflict of interest.
